# Prospective memory assessment: Scientific advances and future directions

**DOI:** 10.3389/fpsyg.2022.958458

**Published:** 2022-07-28

**Authors:** Geoffrey Blondelle, Nicole Sugden, Mathieu Hainselin

**Affiliations:** ^1^CRP-CPO, UR UPJV 72723, Université de Picardie Jules Verne, Amiens, France; ^2^INSPE de l’Académie d’Amiens, Université de Picardie Jules Verne, Amiens, France; ^3^School of Psychology, Charles Sturt University, Bathurst, NSW, Australia

**Keywords:** future, action, experimental task, self-report, informant-report, prospective memory, assessment, memory for intentions

## Abstract

Prospective Memory (PM), the ability to remember to realize intended actions in the future, is crucial for maintaining autonomy. Decades of research has focused on a so-called age PM paradox, where older adults outperformed younger adults on some PM tasks, but not others. Contributing to this paradox is heterogeneity in and a lack of valid assessment methods. Previous research showed a lack of convergent validity between performance-based PM and both self-report and informant-report measures. We argue that questionnaires may be relevant to obtain information regarding patients’ awareness of their PM difficulties but need to be used in conjunction with performance-based tools. Within performance-based PM tools there are also difficulties in measurement: 15–60 min experimental tasks and batteries have a good reliability but cannot usually fit in a standard clinical evaluation, while shorter PM measures have lower reliability and sensitivity. In this perspective paper, we encourage researchers to develop more ecologically valid tools. Innovative PM paradigms that allow participants to generate their own intentions and that take task costs into consideration should be developed. Future research will also need to focus on cognitive factors, personality and online evaluation, to improve PM assessment and develop *ad-hoc* rehabilitation programs.

## Introduction

Prospective Memory (PM) is the ability to remember to realize intended actions in the future (Einstein and McDaniel, 1990). Most research in this field has distinguished between (1) event-based tasks, where actions involve the detection of an external cue (e.g., tell your partner you had a promotion when you see them) and (2) time-based tasks, where actions involve checking the time (e.g., go to the Liverpool stadium at 8:45 p.m. to attend the Champions’ League game). To date literature on PM has brought many tools, theories and models, however there is no consensus on how to assess PM (Ellis, 1996; Burgess and Shallice, 1997; McDaniel and Einstein, 2007). The choice of assessment methods has usually relied on the specific research question (event-based versus time-based) or feasibility and clinical relevance (e.g., patients’ fatigability).

Prospective memory is a crucial factor in maintaining autonomy in daily life (i.e., medical adherence, social, and professional meetings) for many clinical groups (Henry, 2021) and during normal aging (Blondelle et al., 2015, 2016). As such, much research in the last three decades has focused on understanding why younger adults outperform older adults on some PM measures, but not in others.

## Conceptual implications

### A not so paradoxical paradox

During the past three decades, researchers have investigated the Age PM Paradox, as older participants were found to perform worse than young participants in experimental conditions and perform better in ecological conditions (Henry et al., 2004). However, this so-called paradox might not be due to age itself as previously thought, but because of cognitive processes that are differentially affected by aging (Haines et al., 2019).

The so-called Age PM Paradox could be due to the heterogeneity of PM assessments (Azzopardi et al., 2017; Haines et al., 2020; Schnitzspahn et al., 2020). In these studies, deficits in older adults were only found for event-based tasks in the lab, with no age difference for naturalistic event-based tasks. Similarly, benefits for older adults were found for naturalistic time-based tasks, without any age difference for lab time-based tasks. Another important parameter affecting age differences is the source of PM task generation: participants performed better with self-generated intentions rather than experimenter-generated actions to perform. As a result of these findings, now, the so-called age PM paradox is no longer popular and research has shifted its focus toward cognitive factors more likely to explain individual differences in PM, such as executive functions, retrospective memory or IQ (Hainselin et al., 2011; Azzopardi et al., 2015; Koo et al., 2021). Research has also focused on the ecological validity of PM tasks in assessing these factors involved in PM as well as their relationship with self-report measures (Hainselin et al., 2021; Sugden et al., 2021a).

### Assessment of a multidimensional prospective memory construct

#### Assessment of cognitive resource allocation

According to the Multiprocess Framework (McDaniel and Einstein, 2000), PM performance is determined by the allocation of cognitive resources based on the nature of the task (e.g., time-based versus event-based), ongoing task, retrieval cues (e.g., frequency and focality), socio-environmental context (e.g., use of compensatory aids), and individual characteristics (e.g., personality). To capture the multidimensionality of PM, various performance-based laboratory, naturalistic, and virtual reality tasks have been used across studies, which have unsurprisingly produced inconsistent findings across studies. In a meta-analysis, Anderson et al. (2019) synthesized inconsistent findings regarding the effects of performing a PM task in conjunction with an ongoing activity, as opposed to performing the ongoing task alone. They found that increasing the number of non-focal cues in the PM task did not affect PM accuracy but affected speed of responding in the ongoing task (i.e., ongoing task costs), whereas, increasing focal cues affected PM accuracy but did not exert significant costs on ongoing task response times. These findings do not fully support the assumptions of the multiprocess framework, which suggests that increasing the number of focal cues that rely on spontaneous retrieval processes should not affect PM accuracy. Extensions of monitoring theories that have proposed that attentional resources can be flexibly allocated during a PM task in response to ongoing task demands (e.g., the Dynamic Multiprocess Framework and the Preparatory Attentional and Memory model in context) (Scullin et al., 2013; Smith et al., 2017) also do not explain discrepancies resulting from different numbers of PM cues in focal tasks. Given these gaps in existing frameworks, a framework that can consolidate the influence of PM task characteristics on PM performance is needed.

#### What do prospective memory self-reports measure?

Self-report measures of PM differ in the aspects of PM that are measured [see Sugden et al. (2021a) for a review]. The Prospective Memory Questionnaire (PMQ) (Hannon et al., 1995) measures PM lapses and techniques to remember; the Prospective and Retrospective Memory Questionnaire focuses on PM versus RM failures (PRMQ) (Smith et al., 2000); the Comprehensive Assessment of Prospective Memory (CAPM) (Shum and Fleming, 2014) and the Brief Assessment of Prospective Memory (BAPM) (Man et al., 2011) assess forgetting of activities of daily living, with the CAPM also measuring task importance and reasons for forgetting; and the Prospective Memory Concerns Questionnaire (PMCQ) (Sugden et al., 2021b) investigates forgetting behaviors, memory concerns, and retrieval failures. Illustrating these differences in self-report subscales, Cuttler and Taylor (2012) found cannabis users reported more PM failures than non-users on the PMQ internally-cued subscale, but there were no differences on other PMQ subscales or the PRMQ.

In addition to inconsistencies within performance-based and self-report methodologies, an enduring finding is a lack of convergent validity between self-report and performance-based PM measures. In their scoping review of self-report PM questionnaires, Sugden et al. (2021a) found that the relationships between self-report and performance-based PM measures were mostly non-significant and weak to moderate in size. While this may suggest that self-report measures are not valid indicators of PM ability (Uttl and Kibreab, 2011), we contend that poor convergent validity across measurement methods may equally reflect issues with the reliability and validity of performance-based criterion measures.

According to frameworks proposed by Phillips et al. (2008) and built on by Jones et al. (2021), the most ecologically valid tasks are those observing self-generated intentions within the individuals’ everyday environments, such as medication adherence observations (e.g., Woods et al., 2009). Naturalistic experimental tasks such as the envelope task (Huppert et al., 2000) or PM test batteries are deemed slightly less ecologically valid. While many of these tasks permit compensatory aids, and are carried out in neuropsychological or home settings, they are typically single-trial, experimenter-generated, short-interval tasks. These tasks are therefore prone to ceiling effects, restricted scoring ranges, and poor reliability [see Blondelle et al. (2020) for a review of PM measures]. Virtual reality tasks such as the Virtual Reality Everyday Assessment Lab (Kourtesis et al., 2020) involve immersive environments such as shopping precincts. Yet, ecological validity is still inhibited due to the use of experimenter-generated intentions and limiting of strategic aids normally used in everyday PM tasks (Haas et al., 2022). Laboratory-based tasks designed to isolate and control factors underlying PM performance are the least ecologically valid of performance-based measures, due to their use of computerized, experimenter-generated intentions in artificial settings (Phillips et al., 2008). Using this framework of task ecological validity, Jones et al. (2021) found PM intervention studies typically had low to moderate ecological validity, which culminated in few improvements observed in long-term everyday PM in the studies reviewed. Their framework placed self-report measures as the least ecologically valid, due to their low convergent validity with performance-based measures. However, it was acknowledged that self-report measures do still assess performance on ecologically valid tasks and may detect transfer of training to everyday PM tasks. Supporting this, Sugden et al. (2021a) found self-reported PM improvements following open skills interventions (i.e., broader psychoeducation on health behaviors and memory strategies) and performance-based improvements in closed skill interventions (i.e., specific cognitive tasks). As such, self-report measures of PM differ from performance-based measures in that they capture additional psychological and environmental aspects of the multidimensional PM construct.

There is evidence to suggest that variance in self-report PM measures can be explained by factors such as personality (Buchanan, 2017), mood (Steinberg et al., 2013), use of compensatory strategies (Uttl and Kibreab, 2011), and social motivation (Penningroth et al., 2011). Measuring these factors in self-report PM questionnaires provides important insights into individuals’ beliefs, concerns, and goals which can then be used to identify reasons for forgetting and inform treatment strategies (Roche et al., 2002). However, these factors may also confound and provide inaccurate representations of an individual’s PM performance.

Self-reports of PM may be problematic when an individual lacks insight into their PM ability. Individuals may overestimate or underestimate their ability (Cauvin et al., 2019), forget instances of forgetting, or experience neurological impairments that affect metacognitive awareness (Roche et al., 2002). In these situations, informant reports of PM where a proxy observes an individual’s PM performance, may better reflect performance-based PM. However, informant-reports, just like self-reports, may be influenced by factors such as the patient’s age, or the informant’s own levels of subjective burden and distress (Smith et al., 2000; Morrell et al., 2019). Indeed, Sugden et al. (2021a) found in their scoping review that informant-reports had similar non-significant, weak to moderate sized relationships with performance-based measures to self-report measures. Moreover, relationships between self-report and informant-report PM were inconsistent. They concluded that self-report measures of PM may be more accurate in non-clinical populations, but cognitive impairments become more severe, informant reports that rely on observations may provide a more valid report of PM performance. Nevertheless, we recommend further research into variables that influence self-reports and informant-reports of PM.

### Recommendations to improve and expand prospective memory assessment instruments

To date, there is a whole arsenal of tools devoted to the assessment of PM abilities such as paper and pencil test batteries, experimental procedures, single-trial procedures and questionnaires [for review, see Blondelle et al. (2020); see also Table 1 for an overview of the existing PM measures]. This diversity of PM measures and their characteristics shows that no single tool can meet all clinical requirements. PM test batteries and experimental procedures generally have a good reliability as they offer a wide range of trials (between 4 and 8 for PM test batteries and between 18 and 50 for experimental procedures). Such measures are also conceptually relevant as they allow an examination of the main dimensions of PM (event- and time-based). However, they can be difficult to incorporate into a standard neuropsychological assessment because they require significant administration time ranging from 15 to 60 min. In this context, clinicians may prefer the use of single-trials such as the Key task (Babicz et al., 2019) and the Envelope Task (Huppert et al., 2000) due to their low cost and brevity. However, it should be noted that these brief PM measurements have lower reliability and sensitivity compared to test batteries and experimental procedures which may limit their utility, particularly in clinical settings. Because of their structure, the use of such measures does not accurately characterize the nature of PM difficulties encountered by patients. Therefore, the use of single-trial PM measures may be primarily useful for global screening purposes and to demonstrate to patients and their families how difficulties in retrieving intentions may impair autonomy in everyday-life situations. Finally, while questionnaires do not appear to be valid measures of PM performance as measured by laboratory and naturalistic PM tasks, their use may be relevant for obtaining information regarding patients’ awareness of their PM difficulties and for targeting interventions toward areas of greatest concern for the individual.

**TABLE 1:** Overview and brief description of the existing prospective memory (PM) tools.

	Measures	Key features	Time for administration
Test batteries	Rivermead Behavioral Memory Test (RBMT)	Three event-based PM tasks (e.g., “remembering where a belonging is hidden and asking for it to be returned at the end of the test”).	30 mn.
	Cambridge Test of Prospective Memory (CAMPROMPT)	Three time- (e.g., “requesting the newspaper after a twenty-minutes delay” and event-based (e.g., “changing pens after having achieved seven puzzles” PM tasks).	25–30 mn.
	Memory for Intentions Screening Test (MIST)	Four time- (e.g., “remind the clinician to ring the reception when the clock indicated 10 past five and after an interval of 20-min filler task) and event-based (e.g., “switch to another task when there is a question about a former British television program during the general knowledge quiz”) PM tasks + 1 (optional) ecological call-back task with a 24 h delay. Two parallel versions of the test.	30–40 mn.
	Royal Prince Alfred Prospective Memory Test (RPA-ProMem)	Four time- (e.g., “ask the clinician when the session ends today after a 2-min delay”) and event-based (e.g., “writing the name of the attending physician when the clinician shows a form”) PM tasks distributed over short (15 mn to the end of the testing session) and long-term (1 week after the testing session) retention. No classical filler tasks such as puzzles or questionnaires (i.e., tasks selected by the clinician). Three parallel versions of the test.	15 mn.
Single-trial procedures	Envelope task	Single-trial event-based PM task: remember to write a given name and address on an envelope when it is shown and perform some other actions (i.e., add initials, seal the envelope and return it back to the clinician).	10 mn.
	Prompt card task	Single trial event-based PM task: remember to return a card containing information about the next appointment at the end of the testing session.	Total duration of the testing session.
	Telephone test	Single trial time-based PM task: remind the clinical to make a phone call to the counter 5 min after the instruction.	5 mn.
	Key task	Single trial event-based PM task: The clinician informs the patient that an object is going to be hidden (i.e., keys or another object) and the patient must remind the clinician to retrieve the hidden object at the end of the session.	Total duration of the testing session.
Questionnaires	Prospective Memory Questionnaire (PMQ)	Fifty two items related to the frequency of PM difficulties encountered in everyday life (e.g., I forgot to follow a change in my usual routine), measured on a nine-point Likert scale SubScales: Long term episodic; Short term habitual; Internally cued; techniques used to assist recall.	15–17 mn.
	Prospective and Retrospective Memory Questionnaire (PRMQ)	Sixteen items related to the frequency of prospective and (e.g., “Do you fail to mention or give something to a visitor that you were asked to pass on?”) retrospective memory (e.g., “Do you fail to recognize a place you have visited before?”) difficulties encountered in everyday life, measured on a 5-point Likert scale Subcales: Episodic memory (retrospective and prospective); Type of cue (self- or internal cue, i.e., time- and event-based cues); Delay (short- and long-term).	3–5 mn.
	Comprehensive Assessment of Prospective Memory Questionnaire (CAPM)	Thirty nine items related to the frequency (Section A) and degree of concern (Section B, the same items) of PM difficulties encountered in everyday life, measured on a 5-point Likert scale. The two sections include both IADL items (e.g., “Forgetting to buy an item at the grocery store”) and BADL items (e.g.,“Not locking the door when leaving home”). The CAPM also includes 15 additional items related to the reasons of PM failures (Section C; e.g., “When I forget to do something I had planned to do, it is usually because I forgot what I actually had to do?”), measured on the 4-point Likert scale.	13–15 mn.
	Brief Assessment of Prospective Memory questionnaire (BAPM)	Short version of the CAPM with both IADL and BADL items (eight PM items for each).	5–7 mn.
	Prospective Memory Concerns Questionnaire (PMCQ)	Thirty five items related to the frequency of PM difficulties encountered in everyday life, measured on a 4-point Likert scale. Subcales: Forgetting Behavior (e.g., “I forget to pass important messages on to family, friends, or colleagues”); Memory Concerns (e.g., “I forget important dates, birthdays, or anniversaries”); Retrieval Failures (e.g., “forget to do things that I have started such as hanging washing out once the washing machine has finished”).	10–15 mn.
Experimental procedures	Prospective Remembering Video Procedure (PRVP)	Twelve-minute movie recorded in a shopping mall with 21 event-based PM tasks. Participants are asked to write the task to be performed on a piece of paper at the right time in the video.	12–15 mn.
	Test Écologique de Mémoire Prospective (TEMP)	Twenty-min movie simulating real activities of daily living in various areas (i.e., commercial, residential and industrial) with 10 event- and 5-time-based PM tasks. Participants are asked to press a key on a keyboard before recalling the action to be performed.	20–25 mn.
	Virtual Week	Computerized PM tool which simulates daily life activities on a virtual board game. The program offers 10 PM tasks/virtual day. Tasks are distributed according to event- (e.g., “drop the dry cleaning off when you are at the shops”) and time-based cues (e.g., “take your asthma medication every day at 11 a.m.”), task regularity (regular and irregular) and time (time-interval and time of day for the time-based tasks). This tool also provides a detailed overview of the type of errors produced.	15 mn (2 day version) to 1 h (full version).
	Actual Week	Adaptation of the Virtual Week in naturalistic settings in which participants were asked to report on a sheet the PM tasks correctly performed during the day without reconsulting the sheets after completion. The protocol includes a total of 8 PM tasks per day, assigned according to time- (e.g., tasks to be done at lunch time) and event-based cues (e.g., task to be done at a specific time of day) and task regularity.	5 days.

BADL, basic activities of daily living; IADL, instrumental activities of daily living; PM, prospective memory.

In a recent review on performance-based PM assessment instruments, Blondelle et al. (2020) pointed out that most of the PM measures suffer from a lack of adequate normative data, translation into another language, and cross-cultural adaptations. This result is not very surprising, and is consistent with clinicians’ concerns about neuropsychological assessment (Rabin et al., 2005, 2016). For example, the lack of adequate normative data is one of the most challenging issues in the field of neuropsychological assessment for several reasons including substantial heterogeneity on important variables (e.g., sample size, age, gender, education, language, culture, location) and the inconsistent use of non-clinical versus specific clinical populations in standardization studies (Strauss et al., 2006; Puente et al., 2013). Future validation and standardization PM studies should take into account these essential aspects by referring to the guidelines proposed by the International Test Commission (Hernández et al., 2020) to allow a better interpretation of scores and their comparability across cultures.

## Methodological implications

### Developing paradigms that more broadly capture the prospective memory processes involved in everyday life

Over the past three decades, researchers have developed many innovative paradigms and analytical techniques to study PM. These paradigms, techniques, and tasks have been very helpful the predictions of these theoretical approaches (McDaniel and Einstein, 2000; Smith, 2003; Scullin et al., 2013; Strickland et al., 2019), isolating the strategic and spontaneous retrieval processes that contribute to PM remembering [for a review, see McDaniel et al. (2015)] and identifying the vulnerability of certain processes to forgetting. Notwithstanding these advances, we believe that it is essential to continue to develop PM paradigms that are able to capture the processes involved in real-life situations.

In laboratory studies, PM is typically studied using variants of the paradigm initially developed by Einstein and McDaniel (1990) in which participant are required to perform an ongoing task (e.g., lexical decision task) while remembering to generate an alternative response at some time point or upon the occurrence of a specific target event (e.g., press a designated key when the syllable “tor” appears on the screen during a lexical decision task). However, the definition of PM appears to be broad enough to overcome some limitations inherent to the design of most PM tasks. As an example, most everyday event-based PM tasks could have any delay, be performed more or less regularly, triggered by a wide variety of target events, and be related to other individuals. In practice, these aspects are mostly examined in isolation when designing PM tasks, mainly for experimental control and measurement reasons (e.g., researchers often decide to present the same target event several times to improve the reliability of PM tasks), which tends to obscure PM demands in daily life.

While this approach may be of interest for confronting different theoretical positions, it may not be entirely appropriate for understanding the role of PM processes in everyday life and how we manage to remember to take medication and to fulfill our social and professional obligations. However, it is precisely these field questions that have driven research on PM during the last three decades. In natural settings, one of the most key features of PM tasks is that individuals tend to formulate their own intentions in contrast to laboratory tasks where intentions are assigned always by the experimenter. Future studies could develop innovative laboratory PM paradigms that allow participants to generate their own intentions or to choose intentions that are close to those they are asked to perform in a laboratory setting. Some existing flexible PM paradigms, such as the Virtual Week (Rendell and Craik, 2000), may be appropriate candidates to address this challenge by allowing participants to choose activities that are most appropriate for their lifestyles.

### Interpreting ongoing task costs and limitations

One of the most common findings in experimental studies on PM is that the introduction of a PM task produces a detrimental effect on the ongoing activity in which the PM task is embedded [referred to as *ongoing task costs*; Smith (2003)]. In a PM condition, the presence of ongoing task costs is traditionally interpreted as reflecting a change in resource allocation policies away from the ongoing task toward the PM task. As an example, some theoretical positions such as the Preparatory Attentional and Memory Processes theory (Smith, 2003) argue that successful PM performance entails resource-demanding processes which take the form of strategic monitoring to search for relevant environmental cues in order to carry out the intended action. Since monitoring involves attentional capacities that are inherently limited, these are diverted away from the ongoing task, thereby resulting in slower and less accurate responses [for meta-analysis, see Anderson et al. (2019)].

While there has long been a consensus among researchers that costs are true markers of sharing resources between the ongoing task and the PM tasks, more recent research has provided an alternative explanation to this dominant interpretation. For example, the Delay Theory (Loft and Remington, 2013; Heathcote et al., 2015) argues that costs may be due to a tendency for individuals to slow down their responding to give themselves time to accumulate sufficient PM-related information while avoiding being absorbed by ongoing task processing. Scientific advances in mathematical modeling have made it possible, using the diffusion and linear ballistic accumulator model (Brown and Heathcote, 2008), to generate parameters that reflect the processes underlying costs. Such models are useful because they take into account both accuracy and response times, two measures that are typically considered separately when considering ongoing task costs. They also have the advantage of fitting the empirical data well (Heathcote et al., 2015) and account for the anticipatory mechanisms deployed by individuals to deal with the ongoing task demand following the addition of a PM task. By adjusting their decision thresholds, individuals will have more time to discriminate between ongoing task trials and those specific to the PM task.

While the Delay Theory provides insights into the origin of costs by showing that threshold shifts as derived from diffusion and linear ballistic accumulator explain much of variance in ongoing task response times, it does not propose a specific mechanism responsible for PM performance (Strickland et al., 2019). The Prospective Memory Decision Control framework (Strickland et al., 2018) has been proposed to allow for a more comprehensive analysis of task performance including reaction times and accuracy for both the ongoing task and the PM task. The model also allows researchers to study the two classes of cognitive control proposed by Braver (2012) in the context of PM research: (1) the proactive control which is active in advance of a future cognitively demanding event (i.e., the target event) in order to prepare the response and (2) the reactive control that occurs when the target event is encountered in order to produce the intended response. This new model provides a fruitful research avenue for understanding when changes to accumulation rates impact performance of ongoing activities and the intended actions in various contexts.

## Conclusion

### Summary

We now have many PM assessment tools, maybe too many. Performance-based PM tools are heterogeneous regarding duration (15–60 min experimental tasks), psychometric validation and feasibility: batteries have a good reliability but cannot usually fit in a standard clinical evaluation, while shorter PM measures have lower reliability and sensitivity. Questionnaires may be relevant to obtain information regarding patients’ awareness of their PM difficulties and need to be used with performance-based tools.

### Future studies and perspectives

Future research will have to select PM assessment instruments with the best psychometric qualities and cultural adaptation, as recently highlighted in a systematic review (Blondelle et al., 2020). The effects of culture on psychological assessment and the need for culturally appropriate tests are widely discussed in the literature beyond PM. Therefore, the PM research community should work to avoid being another WEIRD (Western, Educated, Industrialized, Rich, and Democratic) research topic (Henrich et al., 2010). In the near future, we will need to use more comprehensive assessment methods, combining experimental and ecological tasks with self- and informant-reports to improve PM understanding and clinical practice. Because of the growing availability of videoconference tools, we will also have to evaluate the impact of PM online evaluation, which seems to be as valid as in person evaluation, but still needs improvement to ensure that everyone, especially minority groups, women and the elderly, can access these services (Zuber et al., 2022).

The next big PM issue to deal with will be how to facilitate improvements of PM skills in the elderly and clinical groups. A recent systematic review highlighted the benefits of mental imagery and external aids to improve PM during aging (Tsang et al., 2021). Moreover, research has found that some smartphone applications, such as voice recorders and reminders, seem to be helpful for patients with Mild Cognitive Impairment (MCI) (Scullin et al., 2022). Future research will need to focus on the effectiveness of these interventions, as well as the methods used to assess intervention outcomes. Similarly to PM assessments, PM improvement strategies and rehabilitation should focus on factors influencing it. Recent research pointed to the role of memory load, likelihood of distraction, working memory, metacognitive confidence, and offloading frequency on PM strategies efficacy (Gilbert, 2015; Ball et al., 2022).

In the same vein, an event-related potential study conducted in the field of cognitive training using validated PM tasks such as the Virtual Week reported an enhancement of PM performances on the task with a use of better self-strategies to support PM remembering in everyday life at the end of the training session. Importantly, this performance improvement was accompanied by an increased performance to real-world PM tasks and activities of daily living (Rose et al., 2015). The event-related potential (ERP) results revealed that the benefit of training was associated with a substantial reduction of an event-related potential component associated with the monitoring of PM cues, suggesting a more spontaneous PM retrieval. These results suggest that laboratory-based PM tasks may be good candidates for supporting interventions to improve PM abilities in daily life.

Together, this research will have implications for how we conduct research into PM, for clinical practice, and will have an impact far beyond, with applications for assessing PM and developing interventions for medical settings (e.g., medication adherence), aviation and education.

## Author contributions

All authors listed have made a substantial, direct, and intellectual contribution to the work, and approved it for publication.
